# Unique heterologous fibrin biopolymer with hemostatic, adhesive, sealant, scaffold and drug delivery properties: a systematic review

**DOI:** 10.1590/1678-9199-JVATITD-2019-0038

**Published:** 2019-11-11

**Authors:** Daniela Vieira Buchaim, Claudia Vilalva Cassaro, João Vitor Tadashi Cosin Shindo, Bruna Botteon Della Coletta, Karina Torres Pomini, Marcelie Priscila de Oliveira Rosso, Leila Maria Guissoni Campos, Rui Seabra Ferreira, Benedito Barraviera, Rogério Leone Buchaim

**Affiliations:** 1 Department of Biological Sciences (Anatomy), Bauru School of Dentistry, University of São Paulo (USP), Bauru, SP, Brazil.; 2 Medical and Dentistry School, University of Marilia (UNIMAR), Marília, SP, Brazil.; 3 Medical School, University Center of Adamantina (UNIFAI), Adamantina, SP, Brazil;; 4 Center for the Study of Venoms and Venomous Animals (CEVAP), São Paulo State University (UNESP), Botucatu, SP, Brazil.; 5 Botucatu Medical School (FMB), São Paulo State University (UNESP), Botucatu, SP, Brazil.

**Keywords:** Fibrin sealant, Snake venom, Thrombin-like enzyme, Cryoprecipitate coagulum

## Abstract

Fibrin biopolymers, previously referred as “fibrin glue” or “fibrin sealants”, are natural biomaterials with diverse applications on health. They have hemostatic, adhesive, sealant, scaffold and drug delivery properties and have become widely used in medical and dental procedures. Historically, these biomaterials are produced from human fibrinogen and human or animal thrombin, and the possibility of transmission of infectious diseases by human blood is not ruled out. In the 1990s, to overcome this problem, a new heterologous biomaterial composed of a thrombin-like enzyme purified from *Crotalus durissus terrificus* venom and a cryoprecipitate rich in fibrinogen extracted from buffaloes *Bubalus bubalis* blood has been proposed. Therefore, a systematic review of studies on exclusively heterologous fibrin sealants published between 1989 and 2018 was carried out using the following databases: PubMed, SciELO and Google Scholar. The keyword used was “heterologous fibrin sealant”. The search resulted in 35 scientific papers in PubMed, four in SciELO and 674 in Google Scholar. After applying the inclusion/exclusion criteria and complete reading of the articles, 30 studies were selected, which formed the basis of this systematic review. It has been observed that the only completely heterologous sealant is the one produced by CEVAP/UNESP. This heterologous biopolymer is proven effective by several studies published in refereed scientific journals. In addition, clinical trials phase I/II for the treatment of chronic venous ulcers authorized by the Brazilian Health Regulatory Agency (ANVISA) were completed. Preliminary results have indicated a safe and promising effective product. Phase III clinical trials will be proposed and required to validate these preliminary findings.

## BACKGROUND

Fibrin sealants are biological materials composed of fibrinogen and thrombin. In the presence of calcium and factor XIII, thrombin converts fibrinogen to soluble fibrin, forming a stable clot and mimicking the final step of the coagulation cascade. Due to its property, this product has indications based mainly on its hemostatic and tissue adhesive properties. Thus, its recent clinical and experimental uses include drug administration and tissue engineering applicability [1].

The use of fibrin compounds as a hemostatic agent occurred for the first time in Germany in 1909 [2]. In the 1940s, it was used as an adhesive in the recovery of injured peripheral nerves through the association of autologous fibrinogen and thrombin [3], and also for skin graft fixation [4,5]. 

Sutures are the conventional technique in surgical procedures; however, due to the formation of fistulas, granulomas, tissue ischemia, and lacerations in some cases, tissue engineering has been stimulating the use of fibrin-based sealants [6]. Thus, these products have been used in current clinical and surgical applications, mainly to approach the edges of the skin, to produce adherence to other tissues and to provide hemostasis [6-9].

Traditionally, fibrin sealants are produced in two ways: using autologous or homologous blood derivatives [10,11]. Autologous sealants use the patient's own blood. Although biocompatible and presenting no risk of infectious diseases transmission, they are not feasible in emergency surgeries [12]. As an alternative, the homologous fibrin sealants produced by a pool of human blood has been used [13]. However, in these cases the literature suggests risks of infectious diseases transmission such as hepatitis, HIV and human parvovirus [14-16].

Therefore, a group of Brazilian researchers from the Center for the Study of Venoms and Venomous Animals (CEVAP) of São Paulo State University (UNESP), Brazil, proposed a new heterologous fibrin sealant (HFS) using a snake venom fraction extracted from *Crotalus durissus terrificus* and a cryoprecipitate rich in fibrinogen obtained from *Bubalus bubalis* buffaloes blood [11,17-19]. 

Snake venoms are composed of several proteolytic enzymes, mainly serine proteases [20-22]. These enzymes act by activating or inhibiting the specific blood factor involved in platelet aggregation, coagulation and/or fibrinolysis [20,23,24]. In *in vitro* experiments, a thrombin-like enzyme from the South American rattlesnake acts similarly to human or animal thrombins transforming fibrinogen into fibrin and forming a robust insoluble fibrin-net [20,22].

The first experimental studies using the HFS produced by CEVAP were performed by Juan et al. in 1995 [25], who presented the preparation procedures and observed good adhesive and hemostatic properties in the repair of nerve injuries in rats. In 1998, Thomazini-Santos et al. [26] showed that the cryoprecipitate extracted from bubaline blood had higher concentrations of fibrinogen and better performance when compared with blood from humans, bovines, ovine or equines. Additionally, they concluded that antifibrinolytic agents were not required to this sealant to achieve successful coaptation of skin surgical edges [27]. Stolf et al. [28] applied for the first time this new sealant in humans as an alternative to conventional suture in the nasolabial region, and described a good adhesive capacity and healing of this material. After these conclusions, the researchers standardized the unique HFS using gyroxin - a serinoprotease obtained from the South American rattlesnake - and a cryoprecipitate extracted from *Bubalus bubalis* buffaloes [9,11].

After these trials, several pioneering experimental studies have been carried out. Currently, the new sealant is being used in several clinical and biotechnological practices, such as in dental surgeries [8,29,30], recovery of injured nervous [31-33], bone repair [34-36], and treatment of chronic venous ulcers [37,38,39].

Thereby, due to its clinical importance and increasing use in several health areas, the aim of this systematic review was to evaluate and compare studies on exclusively heterologous fibrin sealants produced worldwide.

## METHODS

A systematic review of the literature on HFS was carried out in November 2018 from PubMed, SciELO and Google Scholar databases taking into account work conducted between 1989 and 2018. The used keywords were “heterologous fibrin sealant”. The inclusion criteria were the analysis of the title and abstract, in order to identify studies that used only heterologous components in the fibrin sealant. *In vitro* studies and reviews were not selected for detailed analysis, only experimental studies.

## RESULTS

**Figure 1.**
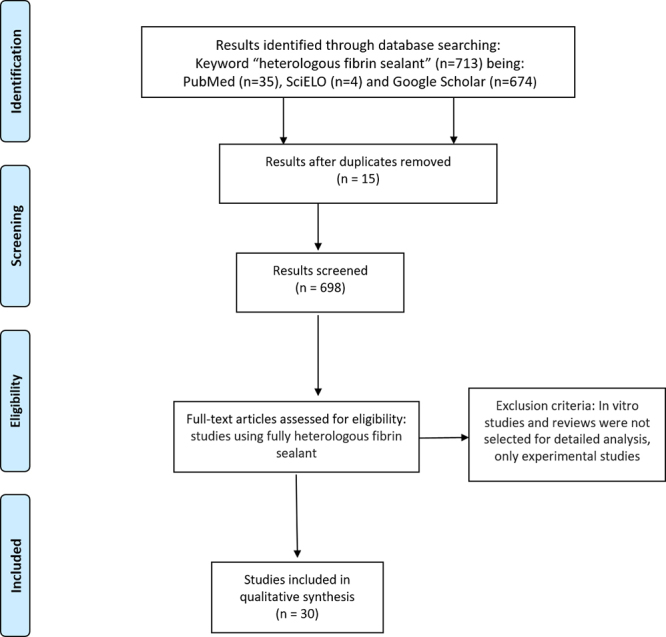
Flowchart of search strategy to identify eligible studies.

The search resulted in 35 scientific papers in PubMed, four in SciELO and 674 in Google Scholar. After applying the inclusion/exclusion and reading criteria, a total of thirty studies were selected to form the basis of this systematic review (Figure 1). Table 1 summarizes the main articles included.

**Table 1.** Summary of articles included in this review.

Author	Objective	Methods	Results	Conclusion	Limitations
**Juan et al. (1995)** [25]	To evaluate the efficacy of fibrin sealant in sciatic nerve repair in rats.	Application of sealant fractions in sciatic nerves of rats. Initially fibrinogen was obtained from human plasma.	The results obtained were similar to those of conventional glue.	The fibrin sealant enabled the adhesion and nervous regeneration.	Lack of data (e.g. number of animals used).
**Sartori Filho et al. (1998)** [40]	Observation of the results obtained with the use of fibrin sealant in sheep testicular biopsy in relation to: hemorrhage control, adhesion prevention, fibrin material effect as a sealing technique. Evaluation of the efficacy of the new fibrin sealant in anastomosis of colon of rats.	Thirty sheep divided into three groups (n = 10). G1: Fibrin sealant group; G2: suture group; G3: control group (without surgery or biopsy).	Twenty days after biopsy - G1: adhesion in three testicles (15%) and two testicles in G2 (10%); G3: without abnormality. On hundred days after the biopsy: orchiectomy. G1 and G2: four testicles (20%) in each group with adherence between the tunics. Subcutaneous adhesions: one (5%) in G1 and three (15%) in G2.	The fibrin sealant proved to be easy to apply, requiring short postoperative monitoring, exhibited fast healing properties and tended to reduce the formation of subcutaneous adherence.	One animal in G2 presented scrotal subcutaneous hematoma and developed testicular degeneration and diffuse subcutaneous adhesions.
**Leite et al. (2000)** [41]	To assess the efficacy of the new fibrin sealant on colon anastomosis of rats.	Eighty rats divided into two groups of 40 animals: G1 (control group): anastomosis with extramucous interrupted suture; and G2: repair suture + fibrin sealant.	Twenty-one days after surgery: G1 exhibited a perfect coaptation of intestinal segments. G2: macroscopic alterations were more intense. There was no difference in the values of force of rupture between the two groups.	Fibrin sealant derived from snake venom proved efficient on allowing anastomosis of the colon of rats.	The use of fibrin sealant in the anastomosis requires further investigation in larger animals to understand the sealant behavior on larger organs.
**Chalhoub et al. (2000)** [42]	To evaluate fibrin sealant derived from snake venom as an alternative to conventional uterine suture after ovine cesarean surgery.	Twenty-eight pregnant sheep divided into four groups of seven animals: six animals were submitted to surgery with fibrin sealant and one animal underwent the conventional hysterorrhaphy.	The healing of the wound showed good macroscopic appearance 30 days after surgery with the application of the sealant. However, microscopically, the uterus did not return to pre-pregnancy conditions 30 days after the experimental cesarean section.	Animals submitted to conventional cesarean section showed a better process of wound healing.	Further studies on the use of this new sealant in hysterorrhaphy after cesarean section in several animal species are required.
**Rahal et al. (2004)** [43]	To assess the effect of snake venom derived from fibrin glue on the viability of split-thickness skin graft in dogs.	One group of nine dogs: a skin graft was collected from the left lateral thoracic area and fixed using several single sutures. On the right side the fibrin sealant + suture was applied.	Compared to the sutured graft, the graft fixed with the sealant showed more cells and a greater number of collagen fibers in the papillary layer of the dermis.	Fibrin sealant increased the survival of the autogenous skin graft, but has moderate adhesive power.	The fibrin sealant did not have sufficient adhesive strength to fix the graft on its own, especially at the site where the wound was induced.
**Moraes et al. (2004)** [44]	Evaluation of the healing process in canine uterus hysterorrhaphy using fibrin sealant derived from snake venom.	Twelve adult female dogs divided into three groups (n = 4). G1: double layer suture; G2: only the fibrin sealant; G3: suture + fibrin sealant as reinforcement.	All groups presented total regeneration of the epithelium, regardless of the treatment. The thickness of healing tissue was higher in G2 than G1 and G3.	The experimental model was appropriate to achieve the proposed objectives. Fibrin sealant produced less inflammation in the exudative phase and eased the evolution of proliferation and maturation phases.	The amount of fibrin sealant used in surgery was not reported.
**Ferraro et al. (2005)** [45]	Assessment of the effect of the fibrin sealant derived from snake venom on the tendon force in the healing process in dogs.	Left and right thoracic limbs of 15 dogs were used. Members were divided into three groups (n = 10). Each group corresponded to the day of evaluation after the application of the sealant: 7^th^, 15^th^ and 30^th^ postoperative day.	There was 62.5% of stump retraction in tendons, and 20.8% moderate to excessive adherence affecting sliding. The group of 30 days showed the best coaptation.	The sealant allowed a progressive increase of the resistance for the maximum tensile force and permanent deformations. Thus, fibrin sealant derived from snake venom can be used to promote healing in the flexor tendon of dogs.	None informed.
**Sampaio et al. (2007)** [46]	To elucidate mechanisms related to the repair of corneal ulcers treated with the fibrin sealant derived from snake venom.	Twenty-one dogs were submitted to lamellar keratectomy divided into seven groups (n = 31). G1 to G6: keratectomized areas with fibrin sealant (collection performed on days 1, 3, 7, 15, 30, 60 after surgery); G7: without sealant.	The corneas collected at day 60 presented discrete changes compared to day 30 in postoperative group. Inflammatory cells were rare, and fibrosis was moderate.	The fibrin sealant helped in repair of the site, contributing to healing mechanisms and preventing the formation of edema in the wound.	Early elimination of fibrin sealant (in three animals) due to the presence of substances in the tear that caused lysis of fibrin.
**Barbosa et al. (2008)** [30]	To compare and to evaluate histological effects in periodontal surgery, especially in gingival grafts, using the new heterologous fibrin sealant compared with traditional sutures, in humans.	Fifteen patients - with surgical procedures in two contralateral areas - were divided into control (only traditional suture technique) and experimental group (fibrin sealant).	At day 14, the experimental group had a more mature epithelial tissue than the control group, but both presented epithelial tissue. At day 45, there were no significant difference between the groups, with an increase in collagen fibers. Epithelium was already mature.	Despite the limitations of the study, the fibrin sealant tested may represent an alternative to sutures in periodontal surgery.	Limited number of patients, sample and statistical analyzes. The histological results were not sufficient to achieve a conclusion.
**Gatti et al. (2011)** [38]	To assess the effect of the new fibrin sealant in the repair process of venous ulcers in 24 adult patients.	Two groups: G1 (control, n = 11) treated with essential fatty acids and Unna’s boot and, G2 (n = 13) treated with essential fatty acids, fibrin sealant and Unna’s boot. Fibrin sealant was applied in the first and fourth week of treatment.	Fibrin sealant was effective in the healing of chronic venous ulcers, presenting ease of application, preparation of the wound bed, reduction of pain and greater number of discharges after eight weeks.	The application of fibrin sealant can improve wound healing processes and increase healing levels.	Preliminary phase I study and major studies with a larger number of patients should be conducted to evaluate the best way to apply the new sealant.
**Iatecola et al. (2013)** [47]	Evaluation of the osteogenic capacity of the new fibrin sealant (FS) combined with bone graft and laser irradiation.	Thirty rats were divided into six groups (n = 5). G1 (control): autogenous graft; G2: graft + laser 5 J/cm²; G3: graft + laser 7 J/cm²; G4: graft + FS; G5: graft + FS + laser 5 J/cm²; and G6: graft + FS + laser 7 J/cm².	The analyses showed absence of inflammatory infiltrate in the bone defect. Bone neoformation occurred in all groups, more intensely in G6.	The osteogenic effect of the 7 J/cm^2^ laser has proved to be very efficient, and its combination with fibrin sealant derived from snake venom can accelerate the regeneration process.	The combination of laser and sealant becomes a therapeutic resource to be further investigated in bone regeneration research.
**Barbizan et al. (2013)** [48]	To assess the effects of fibrin sealant on functional recovery, neuronal survival, synaptic plasticity and glial reaction of the motor neuron after ventral root reimplantation.	Thirty rats divided into two groups (n = 15). G1: unilateral avulsion of the lumbar ventral roots L4-L6; and G2: avulsion followed by reimplantation of the injured root, using the fibrin sealant.	G2 showed no significant changes in the microglial response compared to G1. However, the astroglial reaction was significantly reduced in this group.	The root reimplantation performed with fibrin sealant increased neuronal survival and improved recovery of motor function, improving the regeneration process.	It is not clear whether it is the reimplantation site or the sealant that may be responsible for neuronal survival.
**Benitez et al. (2014)** [49]	Evaluation of motor and sensory functions after dorsal root reimplantation with fibrin sealant associated with mononuclear cells of the bone marrow. Comparison of the local injection of mononuclear cells in the spinal cord with the possibility of homogenizing these cells with the fibrin sealant after avulsion of the ventral root and reimplantation.	Sixty-five rats divided into three groups. G1: rhizotomy (n = 22); G2: rhizotomy + fibrin sealant (n = 23); G3: rhizotomy + sealant + mononuclear cells of the bone marrow (n = 20). Animals submitted to unilateral avulsion of L4-L6 ventral roots divided into four groups. G1: avulsion only; G2: reimplantation with fibrin sealant; G3: reimplantation with fibrin sealant associated with mononuclear cells; and G4: reimplantation with fibrin sealant and injected mononuclear cells.	The reimplantation decreased the glial reaction and improved the synaptic plasticity of the afferent entries. Both replanted groups had twice as much motor control compared to the untreated group. After four weeks: G4 obtained G2-like results; in G3, the neuronal rescue was greater. After eight weeks: G4 with increased degeneration, reaching the level of G1 (injection caused late inflammatory response, occurring delay in cell death); in G3 there was functional recovery, resulting in the preservation of the neurons.	Dorsal root reimplantation with fibrin sealant associated with mononuclear cells of the bone marrow significantly improved motor and sensory function.	The lack of sensory information caused significant motor changes in G1.
**Barbizan et al. (2014)** [32]	To compare the local injection of mononuclear cells to the spinal cord lateral funiculus with the alternative approach of local delivery with fibrin sealant after ventral root avulsion and reimplantation.	Animals submitted to unilateral avulsion of L4-L6 ventral roots and divided into four groups. G1: avulsion only; G2: reimplantation with fibrin sealant; G3: reimplantation with fibrin sealant associated with mononuclear cells; and G4: reimplantation with fibrin sealant and injection of mononuclear cells.	After four weeks: G4 obtained results similar to G2; in G3, the neuronal rescue was greater. After eight weeks: G4 had increased degeneration, reaching the level of G1 (injection caused late inflammatory response, occurring delay in cell death); G3 had functional recovery, resulting in the preservation of the neurons.	The use of the fibrin sealant homogenized with mononuclear cells technique provided the best and most long-lasting results for neuroprotection compared with intra-spinal injection.	The relatively poor clinical outcome requires more attention.
**Cunha et al. (2015)** [34]	Evaluation of the osteogenic potential of a combination of hydroxyapatite (Ha) and the new fibrin sealant (FS) in the improvement of bone regeneration.	Forty rats were divided into four groups (n = 10). Control group: parietal bone defect without treatment; Ha group: bone defect + 8 mg Ha; FS group: bone defect + 8 mL fibrin sealant; and Ha + FS group: bone defect + 8 mg Ha + 8 mL fibrin sealant.	Six weeks after surgery: there was bone growth from the original bone, surrounding several particles of hydroxyapatite, without interposition of the connective tissue. Osteogenesis at the bone defect site was higher in the Ha + FS group.	Hydroxyapatite in combination with the new fibrin sealant accelerated bone repair.	Six weeks were not sufficient for complete repair of the bone defect, requiring more time for skull regeneration.
**Buchaim et al. (2015)** [33]	To assess if the fibrin sealant allows the collateral regeneration of axons from vagus nerve into the interior of a sural nerve graft and if the laser therapy aids in the regeneration.	Thirty-two rats divided into three groups. CG: intact sural nerve (n = 8); EG: one end of the sural nerve graft coapted to the vagus nerve with the sealant (n = 12), and EGL: procedure equal to EG, with addition of low-level laser therapy LLLT (n = 12).	Axonal regeneration observed in EG and EGL. CG: all measured dimensions were larger and with a significant difference in relation to EG and EGL, except for the area and thickness of the myelin sheath, with a significant difference in relation to the EG.	Fibrin sealant was feasible for axonal regeneration, and is an efficient method for recovering injured peripheral nerves. The use of low-level laser therapy has increased nerve regeneration.	In relation to low-level laser therapy, future studies are required to lead to a better understanding of its efficacy.
**Machado et al. (2015)** [35]	To analyze the combination between fibrin sealant (FS) and rhBMP-2 or P1 in the repair of bone defects in rats.	Sixty rats were divided into six groups (n = 10). Control: unfilled bone defect, G2: defect filled with 5 μg of rhBMP-2, G3: 5 μg of P-1, G4: 8 μg of FS, G5: 8 μg of FS and 5 μg of rhBMP-2, G6: 8 μg of FS and 5 μg of P-1.	There was a statistically significant difference (p < 0.05) in all groups after six weeks in relation to the volume of newly formed bones in the surgical area.	The new fibrin sealant proved to be biocompatible and the combination with rhBMP-2 showed greater osteogenic and osteoconductive capacity for bone healing.	The role of fibrin sealants in healing and osteogenesis remains not fully understood.
**Cartarozzi et al. (2015)** [50]	To investigate the effectiveness of mesenchymal stem cells associated with fibrin sealant in the peripheral regenerative process after sciatic nerve tubulization.	Fifteen Lewis rats divided into three groups (n = 5). G1: unilateral sciatic nerve transection followed by implantation of polycaprolactone tubular prosthesis; G2: tube filled with fibrin sealant, and G3: tube filled with fibrin sealant and mesenchymal stem cells.	Sixty days after tubulization, the group with mesenchymal stem cells had a higher myelinated axon counting, more compact fibers and a tendency to increase the thickness of the myelin sheath. Cell treated animals also had better motor function.	The study confirms the efficiency of mesenchymal stem cell treatments after nerve tubulization. In addition, the use of fibrin sealant increases cell reactivity, leading to better compaction of myelinated axons and improving motor recovery.	Although sensory and motor recovery could be detected by stimulating the toes, recovery through gait recovery could not be recorded.
**de Barros et al. (2016)** [51]	To analyze the safety, durability and stability of heterologous fibrin sealant in ovine cartilage repair.	For the implantation of the sealant arthrotomy, chondral defects were induced in eight sheep and divided into two groups (n = 4). G1: euthanasia after seven days, and G2: euthanasia after 15 days.	Seven days after implantation (G1), the sealant was present at the lesion site and stably attached to healthy cartilage. Fifteen days after the procedure (G2), only the lesion site without the fibrin gel was observed.	The applicability of the fibrin sealant was excellent and did not trigger undesirable effects, such as inflammation, allowing a normal repair process in this study.	Further studies are needed, including the use of cell cultures and in vivo comparison with other types of biological sealants.
**de Oliveira Gonçalves et al. (2016)** [36]	Assessment of the effects of low-level laser therapy (LLLT) in stabilized bone graft integration process with a new heterologous fibrin sealant.	Forty rats divided into two groups (n = 20). AFG: osteotomy in the right parietal bone. An extracted fragment was adhered to the left side with the sealant. AFGL: same process, with addition of LLLT.	The AFGL group showed a more evident bone neoformation when compared to the AFG group.	Low-level laser therapy stimulated bone regeneration and accelerated the integration process of autogenous bone grafts.	Fibrin sealant has not yet demonstrated fixing capacity like screws.
**Buchaim et al. (2016)** [52]	To evaluate the effects of low-level laser therapy (LLLT) on the repair of the buccal branch of the facial nerve with two techniques: end-term epineural suture and coaptation with heterologous fibrin sealant.	Forty-two rats divided into five groups. CG: collection of the buccal branch of the facial nerve (n = 10); EGS: with suture and EGF: with fibrin (n = 16). Suture made on the right side and the fibrin sealant on the left side. EGSL: with suture and laser and EGFL: with fibrin and laser (n = 16).	Axonal growth was observed in the distal stump of the facial nerve in all groups. Within ten weeks after surgery, EGSL presented the closest results to CG, in all measured variables, except in the axon area.	Both surgical techniques analyzed were effective in the treatment of peripheral nerve lesions, in which the use of fibrin sealant allowed the manipulation of nerve stumps without trauma. The LLLT presented satisfactory results in facial nerve regeneration.	Despite the satisfactory results, additional studies are required to confirm the effects of laser therapy on peripheral nerve repair.
**Biscola et al. (2016)** [53]	To analyze the viability and efficiency of the end-to-end coaptation of the neonatal sciatic nerve, performed with the application of fibrin sealant derived from snake venom.	Two-day old rats were divided into three groups. AX: sciatic nerve axotomy (SNA) without treatment (n = 30); AX + FS: SNA followed by coaptation with fibrin sealant (n = 30); AX + CFS: SNA followed by coaptation with commercial fibrin sealant (n = 30).	Four weeks post-injury: microglial reaction decreased in the AX + FS and AX + CFS groups. In relation to axonal regeneration, the coaptation allowed the recovery of a greater number of myelinated fibers, with improved morphometric parameters.	Both sealants promote neuroprotection and regeneration of motor and sensory axons. The CEVAP fibrin sealant was easy to handle at the time of surgery.	Because of the small size of rats, combined with the delicate structure of peripheral nerves in the perinatal state, post-transection repair is difficult to perform.
**Vidigal de Castro et al. (2016)** [54]	To compare two fibrin sealants, one derived from human blood and another derived from animal blood and snake venom, in the treatment of axonal lesions, such as ventral root avulsion (VRA).	Animals submitted to unilateral avulsion of the ventral root and reimplantation in the lumbar intumescence (L4, L5 and L6 right side, n = 5 per group). G1: VRA; G2: avulsion and reimplantation with fibrin sealant derived from snake venom (6 mL); and G3: avulsion and reimplantation with commercial fibrin sealant.	Twelve weeks after repair: an improved number of fibers indicating regeneration, regardless of the fibrin sealant used, was observed. Myelin thickness reached values ​​close to normal in the reimplanted groups.	Both fibrin sealants are equally efficient. However, fibrin sealant derived from snake venom is a safer alternative because it is a biological and biodegradable product and does not contain human blood derivatives.	The amount of commercial fibrin sealant used in surgery was not reported.
**Floriano et al. (2016)** [55]	Evaluation of the osteogenic potential of the rubber latex membranes of the clones RRIM 600 and IAN 873 of *Hevea brasiliensis* and the *Hancornia speciosa* latex membranes trough the critical calvarial defect (CSD) model with use of fibrin sealant.	Sixty rabbits divided into two groups, depending on the period of implantation (60 or 90 days), and subdivided into five treatment groups (n = 6). Three groups received natural rubber membrane implants; one was positive control group and one negative control group during each experimental period.	In the 60-day period, a large amount of new immature bone tissue was present in the three groups that received implants. In the positive control, connective tissue was undergoing abundant ossification, indicating the effective formation of new bone and good quality of the newly formed bone.	The fibrin sealant acted satisfactorily, being highly recommended as a substitute for cyanoacrylate in this type of application.	Some membranes were associated with inflammatory reactions in adjacent tissue or were rapidly degraded by the enzymatic activity of macrophages and neutrophils.
**Rosso et al. (2017)** [56]	To evaluate the influence of photobiomodulation therapy (PBMT) on repaired facial nerve lesions through end-to-side suture or coaptation with the new heterologous fibrin sealant.	Thirty-two rats divided into five groups. CG: collection of the buccal branch of the facial nerve (n = 8); ESG and EFG: right side suture and left side sealant (n = 12); ESLG and EFLG: same procedure as before, associated to PBMT with 6.2 J/cm².	A significant difference was observed in the fiber area between EFG and EFLG. In the axon area, fiber diameter, area and thickness of myelin sheath presented no significant differences. The recovery of the vibrissae movement was better in ESLG and EFLG, with results close to CG.	The use of low-level laser photobiomodulation therapy (with suture or fibrin sealant) showed better morphofunctional results.	Future studies could include comparative analyzes between PBMT with new photodynamic therapies, verification of neurotrophic factors and standardization of protocols for laser application.
**Buchaim et al. (2017)** [57]	To assess the efficacy of low-level laser therapy (LLLT) in qualitative, quantitative and functional aspects in the process of facial nerve regeneration after section of buccal branch and suture with fibrin sealant.	Forty-two rats divided into: control group (CG; n = 10): facial nerve collection without lesion; suture experimental group (SEG) and fibrin experimental group (FEG): n = 16, suture performed on the right side, and fibrin sealant on the left side; experimental laser suture group (LSEG) and experimental fibrin laser group (LFEG): n = 16, same procedures as SEG and FEG with laser addition at 6.2 J/cm².	There was a significant increase in the number and density of regenerated axons with laser therapy. In the functional analysis, LSEG and LFEG presented better results in comparison to SEG and FEG.	LLLT increased axonal regeneration and accelerated functional recovery of vibrissae. In both repair techniques (suture and heterologous fibrin sealant), it allowed the growth of axons.	Electrophysiological tests may aid in functional evaluation, which may be considered a limitation for this study.
**Araújo et al. (2017)** [58]	To investigate the neuroprotection provided by human embryonic stem cells modified to overexpress a human fibroblast growth factor (FGF-2), applied with a fibrin scaffold.	Fifty Lewis rats divided into five groups (n = 10). G1: ventral root avulsion and application of fibrin sealant; G2: fibrin sealant, doxycycline and embryonic stem cells; G3: fibrin sealant and doxycycline; G4: fibrin sealant and embryonic stem cells; G5: fibrin sealant and FGF-2 growth factor	The group that received the administration of human embryonic stem cells induced to overexpress the FGF-2 factor by means of doxycycline showed the survival of a significant number of motoneurons when compared to other groups. Embryonic stem cells had a neuroprotective effect, which provided viability to the neurons during the acute post-injury phase.	Transgenic human embryonic stem cells overexpressing FGF-2 in an inducing medium promote neuroprotective effect in the spinal cord after avulsion of the ventral root.	The use of human embryonic stem cells can cause the formation of teratomas.
**Orsi et al. (2017)** [59]	To investigate the citotoxicity and scaffold potential of the association of heterologous fibrin sealant (HFS) with mesenchymal stem cells (MSCs) in the treatment of bone defects in osteoporotic rats.	Twenty rats divided into two groups: non-ovariectomized (NOVX) and ovariectomized (OVX). Four animals from each group were treated with HFS; HFS + MSCs; HFS + MSCs D; four animals were control with lesions; and four control animals without lesions, both without treatment.	Fourteen days after surgery, HFS + MSCs and HFS + MSCs D presented higher bone cell formation at the site compared to the control group (without treatment). Collagen formation was evidenced through bone neoformation in all treated and control groups.	Fibrin sealant was non-toxic to MSCs and showed ability to promote the recovery of bone lesions. In addition, it allowed differentiation of MSCs in MSCs D in the group treated with HFS + MSCs.	It was not possible to observe the total bone repair, since a period of six weeks was not sufficient for the complete recovery of the critical defect in the femur of rats.
**Mozafari et al. (2018)** [60]	To determine the conditions that improve functional recovery after sciatic nerve neurorrhaphy using human embryonic stem cells (hESC) and heterologous fibrin sealant.	A 5-mm segment of the sciatic nerve of mice was removed and rotated 180 degrees to simulate an injury, and the stumps were sutured. Then, the heterologous fibrin sealant and/or hESC was applied at the lesion site.	Sensory function improved when hESCs was used.	The new heterologous fibrin sealant can facilitate nerve repair.	For enhanced functional recovery and better motor neuron reinnervation, fibrin sealant and cell therapy should be used in combination with neurotrophic factors.
**Spejo et al. (2018)** [61]	Evaluation of the use of fibrin sealant as a scaffold to fill the gap formed during induced nerve injury and to retain the stem cells applied at the lesion site.	Group AI (intramedullary axotomy) Group AI + DMEM (intramedullary axotomy + Dulbecco’s modified Eagle’s medium), Group AI + SF (intramedullary axotomy + fibrin sealant), Group AI + CT (intramedullary axotomy + stem cells), Group AI + SF + CT (intramedullary axotomy + fibrin sealant + stem cells).	The groups AI and AI + DMEM, suffered a huge degeneration of the injured motor neurons. The groups treated with fibrin sealant, stem cells and fibrin sealant + stem cells, obtained high levels of motor neurons surviving the induced lesion.	The study demonstrates that mesenchymal stem cell therapy has a neuroprotective activity and, when associated with fibrin sealant, provides a better scaffold to retain cells at the lesion site.	Further studies, including clinical cases, are needed to understand and enhance the recovery of injured complexes.

## DISCUSSION

**Figure 2.**
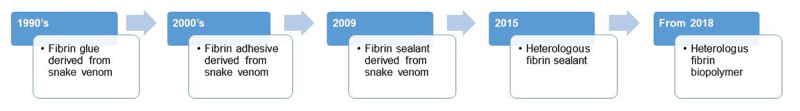
Evolution of the terminology of the heterologous fibrin biopolymer (HFB).

The unique heterologous fibrin sealant (HFS) produced by CEVAP was the only completely heterologous material validated and included in this review. It possesses several advantages such as: fast production process; low cost; potential to act as a scaffold for stem cells and biomaterials, and as a new drug delivery system [9,62]. Moreover, another positive point is its wide applicability in medical, veterinary and dental practice due to the possibility of custom formulation and replacement of conventional sutures. Considering all the properties described for this bioproduct, which go beyond the adhesive capacity, the name “sealant” was reconsidered and it has recently been called “fibrin biopolymer”. In Figure 2, the evolution since the 1990s of the name fibrin glue is summarized.

**Figure 3.**
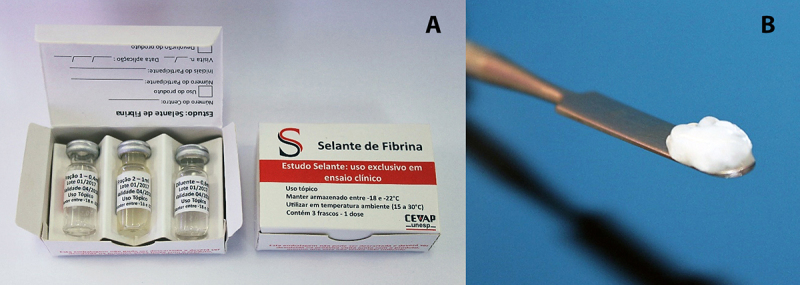
The heterologous fibrin sealant (HFS) produced by CEVAP. **(A)** Fractions, **(B)** stable clot formed after mixture of the components.

Standardized for more than 20 years by Brazilian researchers, the heterologous fibrin biopolymer (HFB) is composed of natural products extracted from animals, without human-blood derivatives (Fig. 3). Therefore, it does not offer risk of transmission of infectious diseases, in addition to possessing low production costs, abundant raw material and possibility of custom formulation to the procedure type [9,11]. The economic impact of this HFB is due to the reduction of surgical time with similar performance or even better when compared to traditional sutures [6,11]. Thus, this systematic review aimed to gather, analyze and evaluate the available evidence on the use of HFB in the period of 30 years in *in vivo* experimental studies. At the end of this, it was verified by the selected studies in worldwide literature research its effectiveness in different clinical applications.

The experimental studies regarding this bioproduct began in 1995 by Juan et al. [25], as previously mentioned, and are being continued to date. Experiments were performed on different organs and animals such as rats, rabbits, dogs, sheep and horses. In this pioneer study, the fibrin sealant was applied in the sciatic nerve of rats, and showed similar efficacy to conventional glue. Also in an animal model, this sealant allowed the coaptation of intestinal segments and proved efficient in obtaining the anastomosis of the colon of rats [41]. However, its use in anastomosis requires additional studies in larger animals to allow analysis in larger organs.

In 1998, Sartori Filho et al. [40] studied its adhesive properties in ovine, using the fibrin sealant and observing their effects on testicular biopsy. The sealant was easily applied, presenting faster repair properties. Its efficacy in hemorrhage control was important to prevent bruising that causes adhesion formation. The advantage over the traditional method (needles and suture lines, for example) was also highlighted, since these was no longer needed.

Chalhoub et al. [42] performed hysterorrhaphy after cesarean sections of sheep. The mortality rate of the animals was 12.5% , which was considered high. As expected, all the operated animals maintained the placenta, which compromised the healing process, having a strong influence on the results of animals in which the sealant was used. Therefore, further studies using this biomaterial on hysterorrhaphy after cesarean section in different animal species are required. 

A third study in ovine was carried out. This time, the implantation of the sealant in chondral defects of cartilage was analyzed [51]. The applicability of the biomaterial was excellent, showed stability and did not trigger undesirable effects such as inflammation, allowing a normal repair process.

In dog models, Moraes et al. [44] assessed the healing process in canine uterus hysterorrhaphy. The results indicate that fibrin sealant may have contributed to moderating the exudative phase, facilitating fibroplasia. In addition, the presence of fibrin in the sealant helps in the formation of a connective base, where the cells can proliferate and form a scar. A complementary study by the same team validated, through biomechanical tests, higher stiffness of the tissue treated with this sealant [63].

Moreover, the study by Rahal et al. [43] sought to analyze the effect of the fibrin sealant derived from snake venom on skin grafts in dogs. The graft adhered to the fibrin sealant showed histological characteristics similar to the sutured graft, but tissue repair of the former was more pronounced, besides having more cells and a greater number of collagen fibers in the papillary layer of the dermis. Its use also had an important effect in reducing the time of surgery, since it minimized the use of suture materials, which is an advantage especially for patients with high surgical risk.

Ferraro et al. [45,64], evaluated the healing strength of the tendon of the thoracic limbs of dogs and their clinical evolution, using fibrin sealant as a substitute for tenorrhaphy. The assessment of four biomechanical properties (resilience, rigidity, maximum limit and stability limit) allowed observing that tendon healing achieved progressive resistance with maximum tensile strength time. Thus, fibrin sealant derived from snake venom obtained positive results, promoting healing in the flexor tendon of dogs [64]. 

Sampaio et al. [46], proposed a model to repair corneal ulcers in dogs. Although fibrinolysis occurred within the first 48 hours, the healing of the eyes treated with fibrin sealant did not present complications. The sealant helped in the local repair, being evident the ease of application and the low cost. 

In the assessment of peripheral nerve recovery, rat models were adopted and fibrin sealant was used for the reimplantation of dorsal spinal roots [49] and ventral [32,54,58,61,65] after its avulsion, contributing in the process of regeneration. Benitez et al. [49] and Barbizan et al. [32,48] also used bone marrow mononuclear cells associated with and homogenized to the fibrin sealant, respectively, which provided more lasting results for neuroprotection, significantly improving motor function. In addition, Cartarozzi et al. [50] found that the application of the association of mesenchymal stem cells and sealant improved the axonal regeneration and reactivity of Schwann cells.

Moreover, Vidigal de Castro et al. [54] compared the treatment of axonal injury between two sealants: one composed of animal blood and snake venom (CEVAP), and another derived of human blood (fibrin sealant homologue). Both sealants were equally efficient, but the heterologous was highlighted as a safer alternative, being a biological and biodegradable product, and without human blood derivatives.

Biscola et al. [53] also compared these two sealants, with the objective of evaluating the end-to-end coaptation of the sciatic nerve in newborn rats. The results were favorable for both sealants, promoting neuroprotection and regeneration of motor and sensory axons. However, the CEVAP fibrin sealant presented greater ease in manipulation at the time of surgery. This corroborates the study by Mozafari et al. [60], in which the fibrin sealant facilitated the repair of the sciatic nerve, allowing adhesion and nerve regeneration. 

Two studies [52,56] evaluated the use of fibrin sealant in the repair of facial nerve lesions with the addition of photobiomodulation therapy. The sealant allowed less manipulation of the nerve stumps compared to the suture, and when combined with the low-level laser, showed better morphofunctional results and nerve regeneration. Buchaim et al. [33] demonstrated that the fibrin sealant was viable for nerve repair. When associated with laser therapy, the sealant showed an even more satisfactory result.

**Figure 4.**
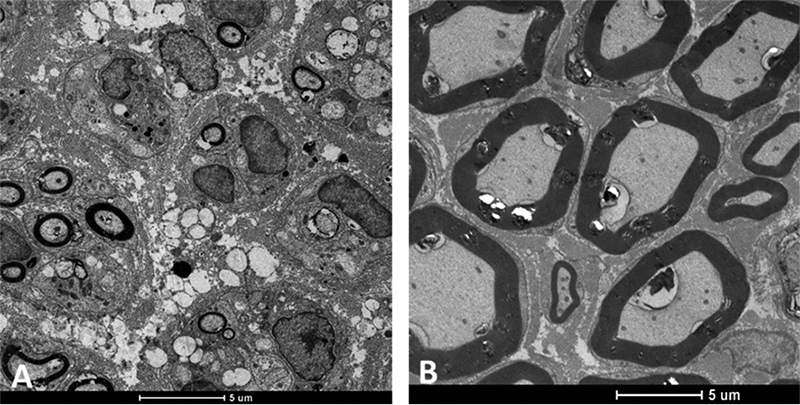
Distal stump fibers of the facial nerve of rats regenerating after end-to-end neurorrhaphy by fibrin biopolymer coaptation. Transmission electronic microscopy: **(A)** 5 weeks post-coaptation and **(B)** 10 weeks post-coaptation.

In the study by Buchaim et al. [57], concerning qualitative, quantitative and functional aspects in the facial nerve regeneration process, the results showed that LLLT increased axonal regeneration and accelerated the functional recovery of vibrissae. Thus, both the suture and the fibrin sealant allowed the growth of axons, and the latter presented good manipulation properties, in addition to the shorter surgery time for nerve repair (Fig. 4). 

The most recent study on nerve reconstruction by Leite et al. [66] validated the use of the fibrin sealant as improvement to suture in sciatic repair, where a protective effect at the lesion site due to the use of HFS was observed.

**Figure 5.**
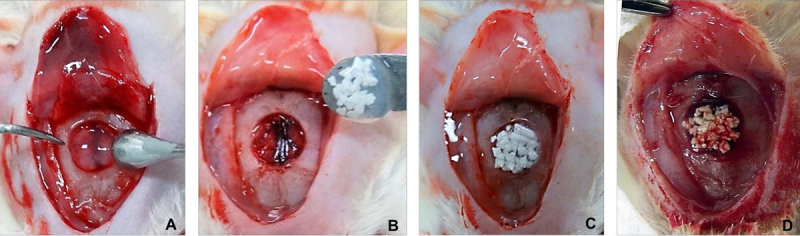
Critical-size defects in rat calvarial bones: **(A)** bioadhesion of the fibrin biopolymer at the edges of the surgical bone cavity; **(B)** osteotomy in the center of the parietal bones and agglutination of the bone graft particles by the fibrin biopolymer before deposition; **(C** and **D)** deposition of the fibrin biopolymer mixture with different bone grafts in the surgical cavity.

In the studies that sought to evaluate the ability of the sealant in relation to bone repair in rats [34-36,47], the osteogenic potential was highlighted. When combined with low-level laser therapy [36,47], or with other biomaterials such as hydroxyapatite [34] and rhBMP-2 [35], the regeneration process was accelerated, proving the biocompatibility of the sealant with this tissue (Fig. 5). 

In order to evaluate the osteogenic potential of the natural rubber membranes of highly bioactive clones of *Hevea brasiliensis* (RRIM 600 and IAN 873) and *Hancornia speciosa*, Floriano et al. [55] used fibrin sealant to correct membranes in bone defects in rabbit calvaria, replacing cyanoacrylate. The sealant proved to be efficient for this type of application, in which the membranes showed good adhesion to the bone surface, and there were no negative responses in the regions where the sealant was applied.

Gasparotto et al. [62] suggested for the first time the use of the new fibrin sealant in vitro as a scaffold for mesenchymal stem cells, being able to maintain cell survival without interfering with differentiation. In addition to the known advantages of its low cost of production and non-transmission of infectious diseases, Orsi et al. [59] evaluated for the first time the performance of the sealant as an *in vivo* scaffold for the transplantation of mesenchymal stem cells in the repair of critical defects of the femur in osteoporotic rats. In that present study, histological analysis revealed that at 14 days after surgery, the animals treated with HFS + MSCs and HFS + MSCs D showed a higher formation of bone cells at the lesion site than the control, suggesting that the sealant served as scaffold. Microscopy electron transmission (MET) analysis showed that fibrin sealant was non-toxic to cells, since the nucleus and cell morphology were very similar to the control group. Therefore, mesenchymal stem cells were proven to promote bone repair when associated with a biological scaffold, showing for the first time non-toxicity to cells. Recently, Cassaro et al. [67] validated the osteogienic potencial of HFS+MSCs on repair of bone defect in femur of rats.

**Figure 6.**
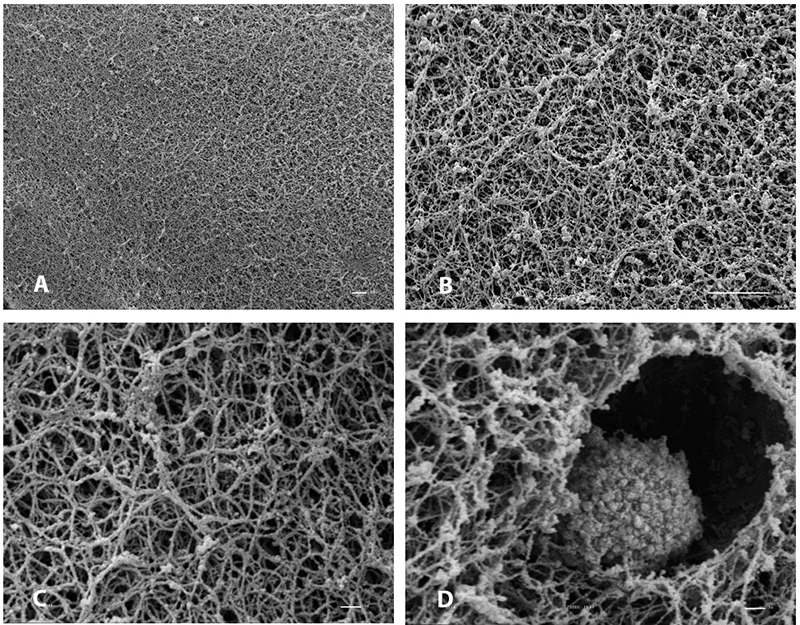
Electron microscopy of the heterologous fibrin sealant at different magnifications. **(A)** 500x; **(B)** 2000x; **(C)** 7000x and **(D)** 7000x with a captured mesenchymal stem cell.

When fibrin is formed after the addition of the cryoprecipitate rich in fibrinogen extracted from buffalo blood, the fibers are randomly formed with spaces between them of different diameters. Figure 6 shows the electron microscopy of the heterologous fibrin sealant at different magnifications and a captured mesenchymal stem cell.

In addition to the application in animal models, clinical trials with the HFB started in 1999 carried out by Stolf [28]. The HFS was for the first time applied in humans as an alternative to conventional suture. Twenty-one Caucasian patients with basal cell carcinoma tumor in the nasolabial region received the product. Skin grafting of the right nasolabial fold was made using HFS, while the left nasolabial fold was sutured. The comparative study of both areas in the same patient showed erythema and edemas on the sutured areas, while dehiscence and serum-hemorrhagic exudation were seen on the glued areas 48 hours after surgery. The cosmetic evaluation of the scar formation was excellent for the glued area and good for the sutured area. The patients did not show any local or systemic adverse events, which makes the new product a valuable alternative method for skin surgery. 

In 2007, Barbosa et al. [29] used HFS in the dental area for the first time. They studied 15 non-smoking patients who needed bilateral gingival grafts around the bicuspid mandibular area. They concluded that the HFS is an alternative to the traditional fibrin adhesive and may represent an alternative to sutures in periodontal surgery [29,30]. In addition, Chiquito et al. [8] compared HFS and conventional suture on gingival defects and considered the first as efficient as the traditional treatment.

Gatti et al. [37,38] used the HFB for the first time to treat chronic venous ulcers assessing its effects on the repair process. They concluded that the application of fibrin sealant may contribute to the wound healing process. Control trials phase I/II will be necessary to evaluate the best way to apply the HFB. In 2015, Abbade et al. [39] used the HFB, previously standardized by Ferreira Jr et al. [9], in a clinical trial phase I/II treating 31 patients. They concluded that HFB was safe for the treatment of chronic venous ulcers according to the proposed dosages. Multicenter clinical trial phase III will be required to establish the definitely efficacy of the product. 

Clinical trials conducted so far show that HFB is a versatile, easy to apply, low-cost preparation, which reduces pain, does not transmit infectious diseases by human blood, and does not present adverse events. Therefore, at this moment the CEVAP team is looking for opportunities and financial support to start the clinical trials phase III and registration by the Brazilian Health Regulatory Agency. 

## CONCLUSION

In conclusion, the studies on different tissue types showed that the fibrin biopolymer is a promising material that achieved the proposed objectives due to the ease of application (reduces surgical time), regenerative properties and biocompatibility with other materials. It is also a safer alternative, since it is a biological and biodegradable product, without human blood derivatives.

As previously mentioned, due to the properties demonstrated in studies that used the heterologous sealant produced by CEVAP, the current denomination was modified to fibrin biopolymer in accordance with other sealants and the literature. In addition to the satisfactory results presented in this review, several studies continue to be conducted to a greater understanding of its performance in different tissues and potential of clinical use.

### Abbreviations

ANVISA: Brazilian Health Regulatory Agency; HFB: heterologous fibrin biopolymer; HFS: heterologous fibrin sealant; MET: microscopy electron transmission.

## Supplementary material

The following online material is available for this article:

Fibrin sealant or fibrin biopolymer: https://youtu.be/y6ho6M0amA8
